# Ultra-secure optical encryption based on tightly focused perfect optical vortex beams

**DOI:** 10.1515/nanoph-2021-0786

**Published:** 2022-01-31

**Authors:** Qingshuai Yang, Zijian Xie, Mengrui Zhang, Xu Ouyang, Yi Xu, Yaoyu Cao, Sicong Wang, Linwei Zhu, Xiangping Li

**Affiliations:** Guangdong Provincial Key Laboratory of Optical Fiber Sensing and Communications, Institute of Photonics Technology, Jinan University, Guangzhou, 510632, China; Department of Electronic Engineering, College of Information Science and Technology, Jinan University, Guangzhou, 510632, China; School of Physics and Optoelectronic Engineering, Ludong University, Yantai, 264025, China

**Keywords:** encryption, gold nanorod, orbital angular momentum, perfect optical vertex

## Abstract

Light’s orbital angular momentum (OAM) with inherent mode orthogonality has been suggested as a new way to the optical encryption. However, the dependence of annular intensity profiles on the topological charge complicates nanoscale light–matter interactions and hampers the ultra-secure encryption application. In this paper, we demonstrate ultra-secure image encryption by tightly focusing perfect optical vortex (POV) beams with controllable annular intensity profiles and OAM states. A simple scheme composed of single spatial light modulator to implement Fourier transform of an ideal Bessel mode with both amplitude and phase modulations is proposed to generate radius-controllable POV in tightly focused beams. Such focused POV beams with identical intensity profiles but varied local OAM density are applied to disorder-coupled gold nanorod aggregates to selectively excite electromagnetic hot spots for encoding information through photothermal deformation. As such, ultra-secure image encryption in OAM states of POV beams in combination with different polarizations can be achieved. Our results lay the ground for diverse nanophotonic applications harnessing the OAM division of POV beams.

## 1 Introduction

Recently, optical vortices have attracted significant research interests owing to the well-defined on-axis orbital angular momentum (OAM) they may carry [1]. A helical wavefront of exp(*ilϕ*) with multiple of 2*π* phase accumulation winding around the beam center denotes its characteristic properties [2]. This prominent feature has impinged diverse photonic applications including optical trapping [3], [4], [5], super-resolution imaging [6], lasing [7], optical communications [8, 9], and metasurface [10], [11], [12]. In particular, the inherent mode orthogonality of OAM states has been suggested as an excellent information carrier for multiplexing to boost the information capacity of both optical communications [13, 14], and holographic encryption [15], [16], [17]. However, the annular intensity profile and peak intensity vary as a function of topological charges of vortex beams. The interdependence complicates nanoscale light–matter interactions with varied both local intensity and OAM density and becomes problematic in applications that require to couple multiple OAM beams into fixed spatial modal distributions.

In this regard, the concept of perfect optical vortex (POV) whose annular intensity profiles of the generated beam are immune to the variation of topological charge has been introduced [18]. An idea POV beam can be treated as the Fourier transform (FT) of a Bessel beam. The general approaches to create such POV beams generally involve the superposition of an axicon phase function with a vortex phase, which has been implemented through metasurface elements [19], digital micromirror device [20], conical axicon [21], and liquid-crystal spatial light modulators (SLMs) [22, 23]. Unfortunately, these methods are only demonstrated effective in paraxial conditions. The presence of an axicon phase leads to defocusing effect in the propagation axis and the shifted focal spot is often understated with degraded both intensity profiles and ring radius, especially in applications where tightly focused POV beams are on-demand.

In this paper, we demonstrate POV with controlled annular radius and peak intensities in tightly focused conditions. The complex field of Fourier transfer of the focused POV expressed as a diffraction-limited annular ring intensity profile superposed with a vortex phase is implemented through a single SLM approach. It allows the generated POV beam with controlled annular radius and arbitrary local OAM densities in the focal plane. As a proof-of-principle, such tightly focused POV beams are applied to gold nanorod aggregates for ultra-secure image encryption in both topological charges and polarizations through a selective photothermal deformation process.

## 2 Results and discussion

### 2.1 Theoretical and experimental verification of tightly focused POV

The complex amplitude expression of an ideal POV with topological charge l is given by [18]:

(1) $$E\left(\rho ,\theta \right)=\delta \left(\rho -\rho_{0}\right)\text{exp}\left(il\theta \right)$$

where (ρ,θ) are the polar coordinates in the beam cross section, δ(x) is Dirac δ-function, and $\rho_{0}$ is the radius of POV. The POV with this complex amplitude distribution can be obtained using the FT property of an ideal Bessel mode, which is given by [24]:

(2) $$B\left(r,\varphi \right)=J_{l}\left(\alpha r\right)\text{exp}\left(il\varphi \right)$$

where α is the wave vector in the transverse direction, $J_{l}\left(\alpha r\right)$ is an lth order Bessel equation of the first kind. In experiment, if we want to generate a POV by means of Eq. (2), it must be bounded by a circular aperture of radius *R*,

(3) $$B_{1}\left(r,\varphi \right)=circ\left(\frac{r}{R}\right)J_{l}\left(\alpha r\right)\text{exp}\left(il\varphi \right)$$

where

$$circ\left(\frac{r}{R}\right)=\left\{ \begin{array}{l} 1,r<R,\\ 0,r>R. \end{array}\right.$$

For the field distribution in Eq. (3), its FT in polar coordinates is given by [22]:

(4) $$\begin{array}{l} U\left(\rho ,\theta \right)={\left(-i\right)}^{l+1}\left(\frac{k}{f}\right)e^{il\theta }\overset{R}{\underset{0}{\int }}J_{l}\left(\alpha r\right)J_{l}\left(\frac{kr\rho }{f}\right)rdr\\ ={\left(-i\right)}^{l+1}\left(\frac{kR}{f}\right)e^{il\theta }\times \left[\frac{\alpha J_{l+1}\left(\alpha R\right)J_{l}\left(XR\right)-XJ_{l}\left(\alpha R\right)J_{l+1}\left(XR\right)}{\alpha^{2}-X^{2}}\right] \end{array}$$

where X=kρ/f, k=2π/λ is the wavenumber, λ is the wavelength of the incident beam, f is the focal length of an ideal FT lens. Using the orthogonality of Bessel functions to simplify Eq. (4), it can be seen that the maximum radius of the focus ring is the same as Eq. (1), being independent of the topological charge. It is worth mentioning that the physical implementation of Eq. (4) by optimal phase modulation only fails to produce POV without undesired side lobes under nonparaxial focusing conditions which are normally requested for high-density information encryption [25]. Therefore, the FT effect of the high-NA objective of the weighted field must be taken into considerations when implementing Eq. (4) under tight focusing conditions [26]:

(5) $$\begin{array}{l} E\left(x,y,z\right)=FT\left\{G\left(\Theta ,\phi \right)\right\}\\ =FT\left\{P\left(\Theta \right)E_{t}\left(\Theta ,\phi \right)\exp\left(jkz\;cos\;\Theta \right)/cos\;\Theta \right\} \end{array}$$

where FT{·} represent the FT; P(Θ) is the apodization function; Θ is the converge angle, which is related to the aperture radius, the numerical aperture and refractive index of the immersion medium; $E_{t}\left(\Theta ,\phi \right)$ is the transmitted field. Then, when the incident optical beam is modulated into the field of Eq. (5), the electric field distribution in the focal plane can be written as:

(6) $$FT\left\{U\left(x_{0},y_{0}\right)\times G\left(\Theta ,\phi \right)\right\}\propto \text{exp}\left(-\frac{{\left(\rho -\rho_{0}\right)}^{2}}{\Delta \rho^{2}}\right)\text{exp}\left(il\psi \right)$$

where $\rho_{0}$ and Δρ denote, respectively, the radius and width of the annulus, and ψ is the azimuthal angle in the focal plane of the objective.

**Figure 1:**
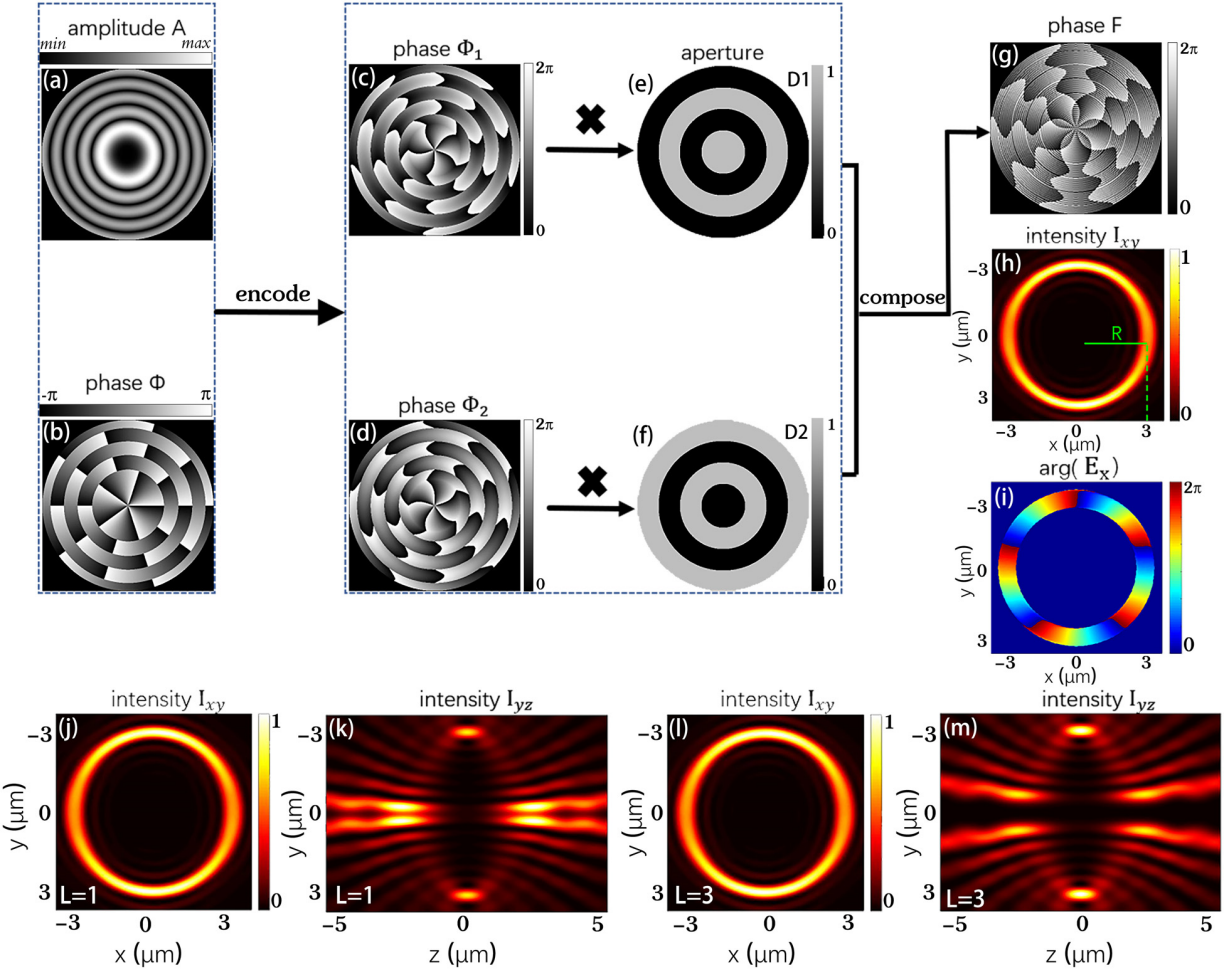
Schematic diagram of the calculation process of perfect vortex phase patterns. (a) Amplitude and (b) phase distributions of the complex field in Eq. (4). (c) and (d) Two phase-only distributions correspond to Eq. (7). (e) and (f) A pair of complementary ring-shaped apertures. (g) The final synthesized phase distribution. (h) and (i) The intensity distribution in the xy cross-section and the phase whose topological charge l=5. (j)–(m) are the intensity distributions in the xy and yz cross-sections, and the corresponding topological charges l=1 and l=3. Simulated parameters of tightly focused system: *x* polarization, λ = 800 nm, NA = 0.95, radius *R* = 3 μm.

As can be seen from Eq. (6), the topological charge will not affect the amplitude distribution, which satisfies the definition of POV. Therefore, we can generate a tightly focused POV by implementing FT of an ideal Bessel mode with both amplitude and phase modulations according to Eq. (4). Now, the complex optical field can be expressed as $U\left(x_{0},y_{0}\right)=A\left(x_{0},y_{0}\right)\text{exp}\left(i\phi \left(x_{0},y_{0}\right)\right)$ in the Cartesian coordinate with the amplitude and phase distributions shown in Figure 1(a) and (b). In this case, the intensity radius R=3 μm, and the topological charge l=5.

To simplify the experimental configuration, we can use a phase-only SLM to generate complex amplitude modulations, in which amplitude and phase are modulated simultaneously. First, we rewrite the complex field expression as the superposition of two phase-only modulations: $U\left(x_{0},y_{0}\right)=A_{max}/2\;\exp\left(i\phi_{1}\left(x_{0},y_{0}\right)\right)+A_{max}/2\text{ exp}\left(i\phi_{2}\left(x_{0},y_{0}\right)\right),$ where the specific expressions are:

(7) $$\left\{ \begin{array}{l} \phi_{1}\left(x_{0},y_{0}\right)=\phi \left(x_{0},y_{0}\right)+arccos\left[A\left(x_{0},y_{0}\right)/A_{max}\right]\\ \phi_{2}\left(x_{0},y_{0}\right)=\phi \left(x_{0},y_{0}\right)-arccos\left[A\left(x_{0},y_{0}\right)/A_{max}\right] \end{array}\right.$$

The corresponding calculation results of two phase-only distributions are displayed in Figure 1(c) and (d). Then, in order to use a phase pattern to generate the complex field, the two kinds of different phase information must be superimposed on a single pattern. Here, we designed a pair of complementary ring-shaped apertures, $D_{1}\left(x_{0},y_{0}\right)D_{2}\left(x_{0},y_{0}\right)$, to combine the two different phase distributions. As shown in Figure 1(e) and (f), they are binary structures with alternating amplitude of 0 and 1, in which each ring has the same width. Superimpose the previously obtained phase distribution through complementary ring-shaped apertures:

(8) $$F\left(x_{0},y_{0}\right)=\phi_{1}\left(x_{0},y_{0}\right)D_{1}\left(x_{0},y_{0}\right)+\phi_{2}\left(x_{0},y_{0}\right)D_{2}\left(x_{0},y_{0}\right)$$

The superimposed phase is displayed in Figure 1(g). Based on the synthetic phase, we can realize the complex optical field. Finally, a POV is created in the tightly focus system.

Figure 1(h) shows the intensity distribution in the focus plane of a high NA objective lens when the incident optical field is modulated by phase F. It can be seen that the intensity profile of the optical field is a ring shape, and the radius is consistent with the theoretically designed value, indicating that the radius is controllable by this method. Simultaneously, its phase distribution of Ex exhibits 5-fold of 2*π* phase accumulation winding around the beam center, coinciding with theoretically designed topological charge l=5 (see Figure 1(i)). Figure 1(j) and (l) show the intensity distributions of POV with the same *R* on the xy cross-section, whose topological charge are l=1 and l=3, respectively. Their intensity distributions on the yz cross-section are shown in Figure 1(k) and (m). We can see that a large part of intensity is located at the focal plane (*Z* = 0) with non-negligible side lobes distributed at defocused planes. Nevertheless, the focal intensity ring with perfect vortex field dominates when the topological charge increases.

**Figure 2:**
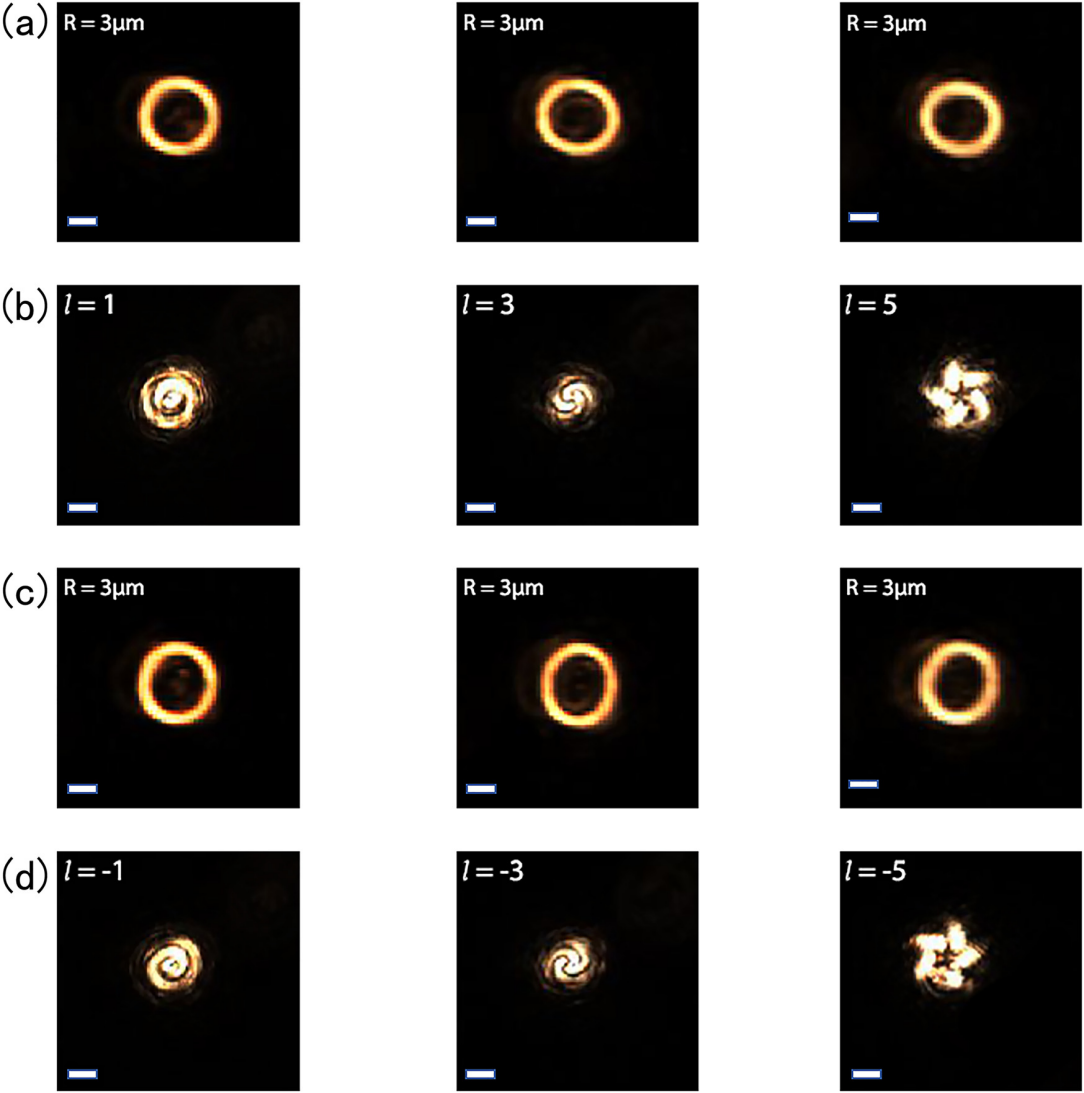
Intensity distributions and corresponding interference patterns of POVs with different topological charges. (a) and (c) Intensity patterns of POV with different topological charges on the focal plane (*Z* = 0). (b) and (d) The corresponding coaxial interference patterns. Scale bar: 3 μm.

To experimentally verify the tightly focused POV, we conducted an interference experiment with our homemade setup. Figure 2(a) and (c) shows the POVs generated on the focal plane of the objective lens, with various topological charges (l=±1,±3,±5) and the same radius (*R* = 3 μm). Then we manage to let another unmodulated Gaussian beam interfere with the POV to confirm the vortex nature of the ring beam [22]. Figure 2(b) and (d) shows the result of the interference experiment, and the spiral fringe pattern confirms the presence of OAM in the POV. The number of fringes represents the topological charge l, and its direction of rotation decides the sign. These results solidly confirm that the tightly focused POV can have the same radius while with controllable OAM states. Furthermore, we show that this method can generate POVs with different radii while keeping the topological charge constant (Supplementary Figure S1).

### 2.2 Simulation and experimental results of optical storage

**Figure 3:**
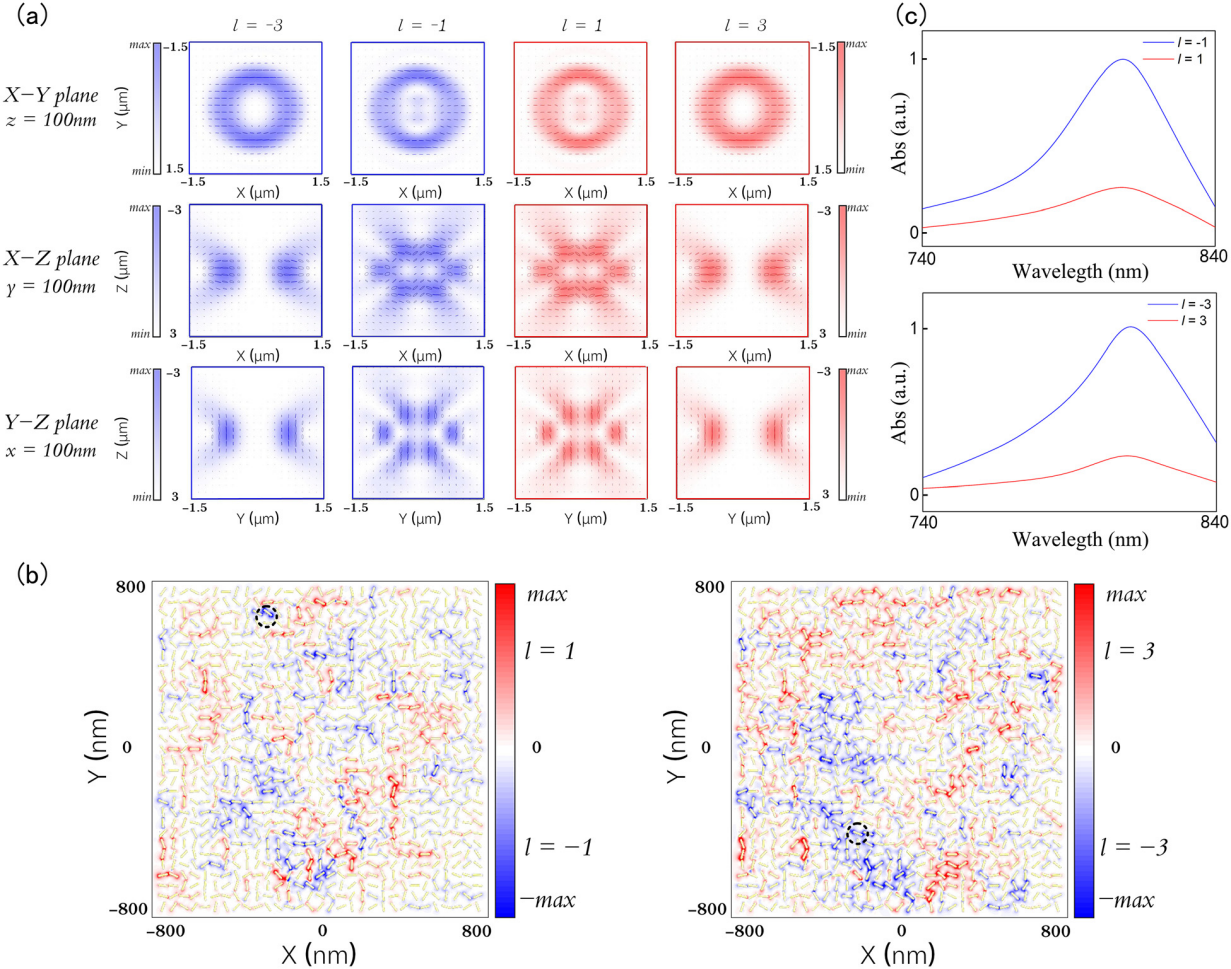
Synthetic helical dichroism by focused POVs. (a) The polarization ellipse and normalized intensity distributions of the xy, xz, yz cross sections in the focal region. The red and blue ellipses represent the polarization state of the points in the focal regions when l=−3,−1,1 and 3, respectively. (b) Distribution of FDTD simulated normalized electric field intensity |*E*|^2^ at the center plane of the array excited by POV beams with opposite topological charges. The red and blue color denote the case of tightly focused POV beams with (l=±1,l=±3). (c) The normalized linear optical absorption difference inside the GNRs near the hotspots in the marked area in (b) excited by tightly focused POV beams with opposite topological charges.

When these tightly focused POV interact with gold nanorod aggregates, the focal polarization distributions play an important role. It has been shown that the vortex beam can introduce OAM-dependent spatial rotations of the focal polarization ellipses and hence synthetic helical dichroism (HD) in individual gold nanorods [27]. Based on the vectorial diffraction integral theory, we can calculate and analyze the electric field components, Ex Ey Ez, of the focal electric field, and further express the polarization state of each point through the polarization ellipse. The polarization ellipse of such focused POV in the xy cross section can be expressed as [28]:

(9) $${\frac{{E_{x}}^{2}}{{E_{0x}}^{2}}+\frac{{E_{y}}^{2}}{{E_{0y}}^{2}}-2\frac{E_{x}}{E_{0x}}\frac{E_{y}}{E_{0y}}\text{cos}\left(\psi \right)=\left(\text{sin}\left(\psi \right)\right)}^{2}$$

where $E_{x}=E_{0x}e^{-i\omega t+ikz+i\delta_{x}}$ and $E_{y}=E_{0y}e^{-i\omega t+ikz+i\delta_{y}}$ are the complex amplitude expression of electric field components; $\psi =\delta_{x}-\delta_{y}$ is the phase retardation between $E_{x}$ and $E_{y}$. Following Eq. (9), the ellipse orientation angle and eccentricity at each point within the cross-section plane can be calculated. Then we superimpose and display the polarization ellipse and intensity distribution corresponding to each point on the defocused plane (*z* = 100 nm), as shown in the first row of Figure 3(a). The calculation parameter is the same as the previous setting, and we select 24 columns of data at equal intervals to calculate the corresponding polarization ellipse. Similarly, we can calculate the polarization states distribution in the yz and xz cross-section planes and the polarization ellipses on the yz and xz cross sections can also be represented by the corresponding $E_{y}E_{z}$ and $E_{x}E_{z}$ components, as shown in the second and third rows of Figure 3(a). It is seen that the orientations of polarization ellipses manifest a spatial dependence. In addition, the rotation of the polarization ellipse is dispersed at the same position of the focal region which is determined by the topological charge of the tightly focused POV (Supplementary Figure S2). Consequently, the POVs with controllable radius and different OAM states can be applied to gold nanorod aggregates to excite localized electromagnetic hotspots to encrypt different information.

In order to provide an intuitive illustration, we used a simplified model to qualitatively evaluate the response of a disordered gold nanorod assembly under the excitation of a tightly focused POV [27, 29]. The simplified model consists of 961 gold nanorods, which are 50 nm in length and 12 nm in diameter. The gold nanorod array contains 31 × 31 randomly oriented gold nanorods whose centers form a square array with a lattice constant of 50 nm. The electric field distribution of gold nanorods can be calculated by the finite-difference time-domain (FDTD) method and the non-uniform grid with the maximum mesh step of 4 nm as well as perfectly matched layer boundary conditions are used in the numerical simulation. By calculating the intensity distribution generated by the interaction between the source which has been imported into the POV’s electric field distribution and the disordered gold nanorods model, we can intuitively see the excitation of localized electromagnetic hot spots by POV with variant topological charges, as shown in Figure 3(b). It is clearly seen that the recording beam with different topological charges can excite different hot spots constituted by different gold nanorods denoted by red and blue color. Then, the GNRs near the hotspots exhibiting strong difference in excitation strength in the disordered arrays were selected to unveal the linear absorption differences to the incident POV beams of opposite topological charges, which is shown in Figure 3(c). As a result of the non-trivial difference in excitation strengths, synthetic HD spontaneously emerges in which the GNR exhibits different linear absorptions for OAM beams [27]. When raising the beam power, the gold nanorods nearby these hot spots can be selectively photothermally reshaped to provide a mechanism for optical encryption in the OAM states of the POV.

**Figure 4:**
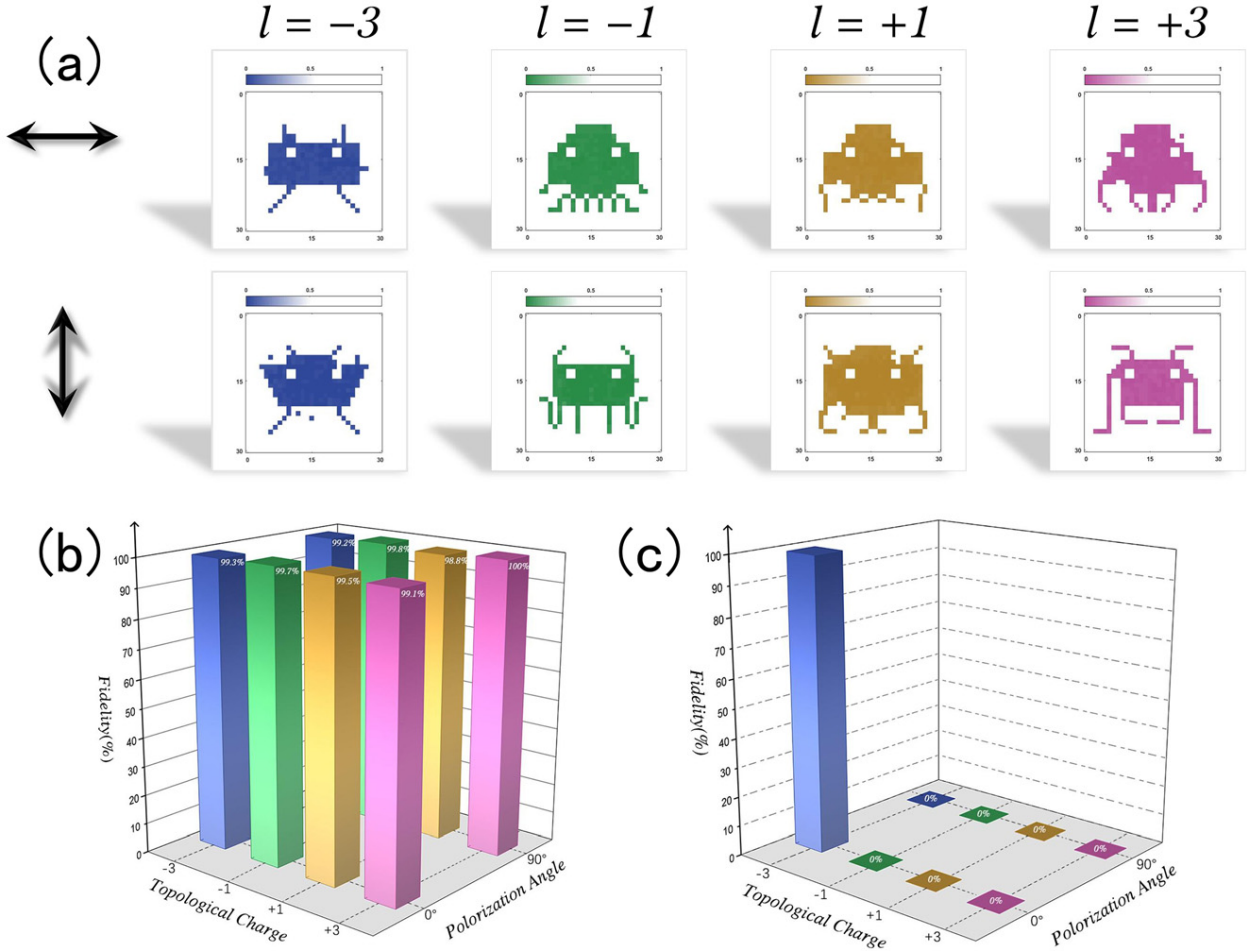
Ultra-secure optical encryption and encoding. (a) Schematic of the optical image encryption in a spatial region (45 × 45 μm^2^ with 30 × 30 pixels). The eight images are the retrieved results using eight different polarization and topological charge combinations, which are outlined in (b). The fidelity of these eight optically encrypted images in (a). (c) Analysis of fidelity of retrieved images using different combination of polarizations and topological charges.

To experimentally demonstrate ultra-secure optical image encryption by tightly focused POV, we prepared samples of gold nanorod aggregates. Gold nanorods with an initial optical density of 90 were mixed with 10 wt% poly(vinyl alcohol) polymer and self-dried at room temperature. In Figure 4(a), we show eight images encrypted at the same spatial region by the eight types of combination with polarization and topological charge, which have been marked in the corresponding positions. Each image consists of 30 by 30 pixels with a pixel separation of 1.5 μm. The images were encrypted through photothermal reshaping of gold nanorods in a homebuilt optical setup (Supplementary Figure S3). The exposure time for each pixel was 20 ms and the laser power used for encryption was optimized to minimize the cross-talk. The recorded patterns were retrieved through the contrast in nonlinear upconversion luminescence [30, 31] between encrypted and non-encrypted regions. On the basis of normalizing the fluorescence intensity, we select suitable thresholds according to the bit error rate (Supplementary Figure S4) to binarize the image, as shown in Figure 4(a). Figure 4(b) presents the fidelities of retrieved images which are calculated based on the raw nonlinear upconversion luminescence data. It can be seen that when retrieving the encrypted images by the POV with identical OAM states and polarizations to the encrypting beams the fidelity can be far exceeding 99%. The wavelengths of nonlinear upconversion luminescence from the gold rod samples acquired in our experiments range from 450 nm to 680 nm (Supplementary Figure S6).

Furthermore, we used a POV beam with topological charge l=1 and polarization P=0° to record an image, and then point-by-point scan the nonlinear upconversion luminescence signal of the recording area with a POV beam of different combinations of polarizations and topological charges. The corresponding fidelity is shown in Figure 4(c). It is clearly seen that the encrypted information can only be retrieved when read by the POV with the same parameter that was employed during the encryption, otherwise the information is read out as noise with fidelity approaching null (Supplementary Figure S5). These results indicate that ultra-secure optical image encryption by the generated POV beams with controllable OAM states and polarization is feasible. Beside, we found that although the perfect vortex beam does not greatly improve the pixel resolution and multiplexing crosstalk compared with the vortex beam, the controllable focusing radius still endows it with new advantages, such as *in-situ* storage, encryption, and laser processing, etc.

## 3 Conclusions

In summary, we propose a novel method to generate POV in the focal plane of tightly focused systems and we can control the radius and the topological charge based on a phase-only formula. Based on the complex expression of the FT of the ideal Bessel beam, we use a single phase-only SLM to achieve a complex field and focus this field onto the focal plane where the created POV is used to encryption. The image encryption has been demonstrated through the interaction between focused POV and disorder-coupled gold nanorod. Additionally, this novel approach can be used to generate other types of POV with different shape of the intensity distribution, such as elliptic POV [32, 33], fractional POV [34]. In theory, the physical dimension of OAM has boundless orthogonal states. It is anticipated that the combination of OAM and other physical dimensions can further increase the storage capacity of optical memory technology. We envision its huge potentials of application of this degree of freedom in different fields, such as multiplexed data storage, optical communication, and quantum entanglement, etc.

## Supplementary Material

Supplementary Material
