# Supplementary material for: Ultra-secure optical encryption based on tightly focused perfect optical vortex beams
Source: Nanophotonics. 2022 Jan 31;11(5):1063–70. doi: 10.1515/nanoph-2021-0786 (PMC11501186; doi:10.1515/nanoph-2021-0786)
Supplement: Supplementary file 1 — Supplementary Material [file j_nanoph-2021-0786_suppl.docx]

Supporting Information

**Ultra-secure optical encryption based on tightly focused perfect optical vortex beams**

*Qingshuai Yang, Zijian Xie, Mengrui Zhang, Xu Ouyang, Yi Xu, Yaoyu Cao, Sicong Wang, Linwei Zhu, and Xiangping Li**

1. Intensity patterns of the generated perfect optical vortex beams with different radius

In **Figure S1**, we present POV with different radius which could be realized by modifying the parameters of the phase-only formula. The corresponding coaxial interference patterns obtained by making the POV interfere with Gaussian beam. The number of spiral fringes is the same as the topological charge, and the shapes will change with the radius.


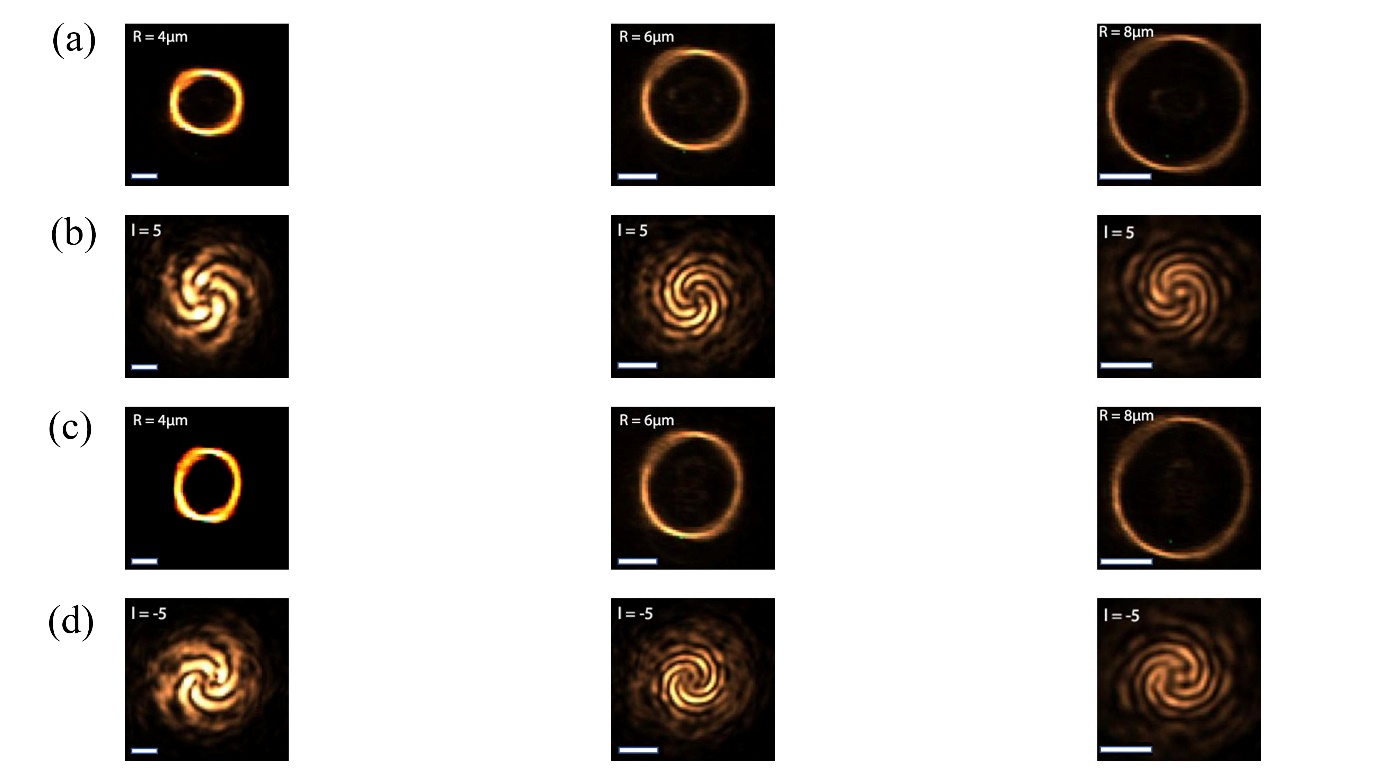


**Figure S1.** a) and c) are the POV’s intensity distributions in the focal plane with various radii (R = 4μm,6μm,8μm), corresponding to $l=5$ and $l=-5,$ respectively. b) and d) are interference patterns of perfect vortex beam and Gaussian beam. Scale bars in three columns denote: 4 μm, 6 μm, 8 μm.

1. The inclination of polarization ellipses on the $\boldsymbol{xy}$、$\boldsymbol{xz}$、$\boldsymbol{yz}$ cross sections

In **Figure S2**(a), we show a schematic diagram of the inclination of the polarization ellipse, which represents the angle ($\theta$) between the major axis of the ellipse and the horizontal axis. The expression of the inclination angle ($\theta$) can be defined as:

$tan2\theta= \frac{2E_{0x}E_{0y}cos\psi}{{E_{0x}}^{2}-{E_{0y}}^{2}}$ (1)

Where $\psi$ is phase difference between $E_{x}$ and $E_{y}$; $E_{0x}$ and $E_{0y}$ are the amplitudes of $E_{x}$ and $E_{y}$ , respectively.

We have shown the polarization ellipse distributions in the corresponding sections in Figure 3(a). To highlight the difference of electric fields with topological charge of different symbols and values, we plot the inclination of polarization ellipses corresponding to different sections, as shown in Figure S2(b). It is clearly seen that there are obvious changes between the cross sections in the electric field of different topological charges and the calculation result has a variation range of 180 degrees, which is consistent with the definition.


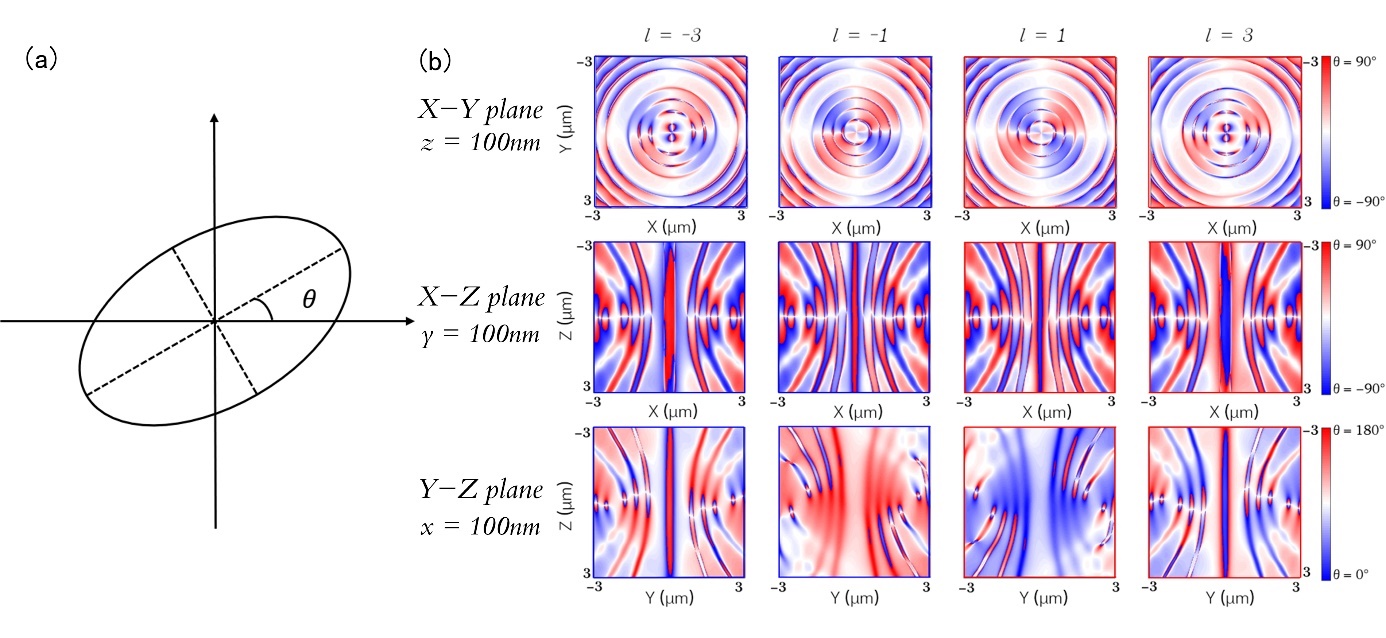


**Figure S2.** a) Schematic diagram of the ellipse inclination $\theta$. b) The distribution of inclinations $\theta$ on the $xy$、$xz$、$yz$ cross-sections, which are offset by 100nm from the focal point of the light field.

1. The optical setup

We perform the optical encryption experiment with our homemade setup shown in **Figure S3**. The 800nm fs laser from a Ti:sapphire oscillator (Chameleon, Coherent) is expanded and collimated onto a half-wave plate and a Glan prism to adjust the power of laser and control the direction of polarization of the outgoing light. The output light oblique incident into the phase-only space light modulator (HOLOEYE, Pluto LC-R2500) and then through the 4F system and dichromatic mirror (FF735-Di02-25 × 36, Semrock). Finally, the laser focused to the sample placed on a three-dimensional stage (P-563.3CD, Physik Instrumente) by an objective. The nonlinear upconversion luminescence of the sample was collected by the same objective and detected by an avalanche photo-diode (SPCM-AQRH-14-FC, Excelitas Technologies), which is placed in the position of the CCD in the diagram.


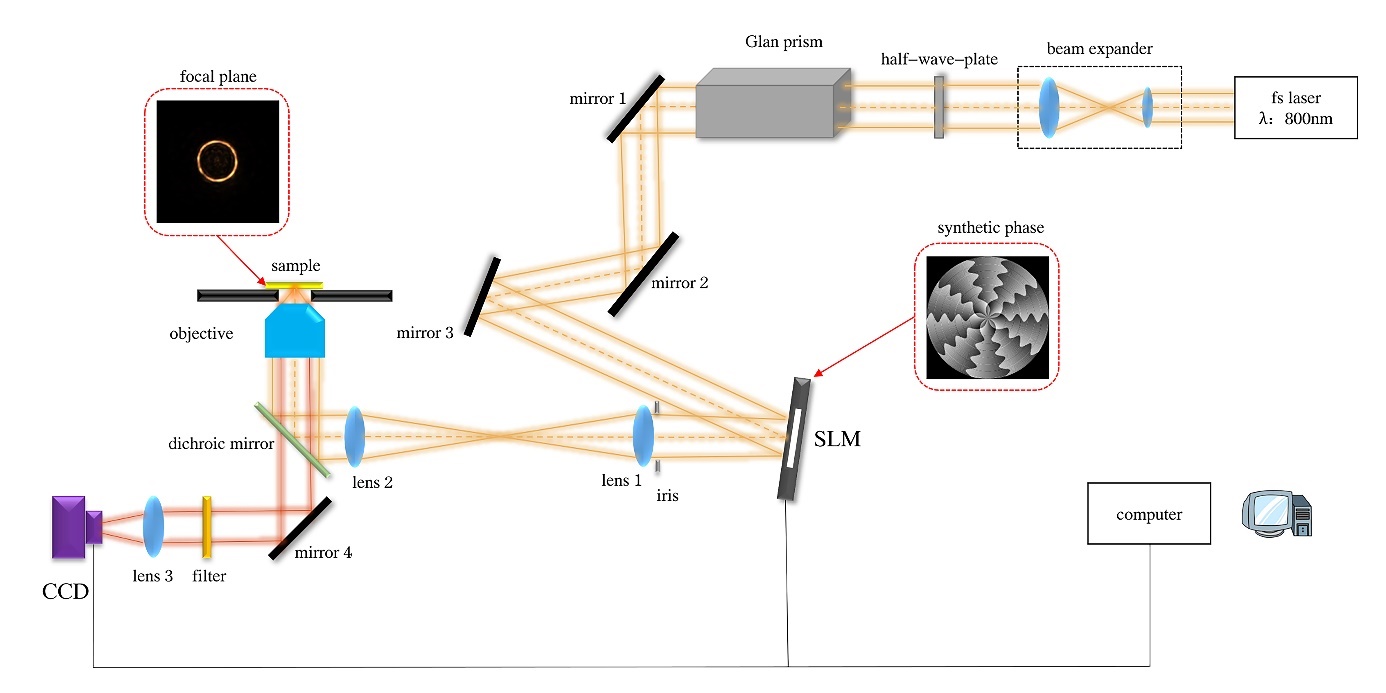


**Figure S3.** The schematic of the optical setup used in the optical encryption experiment. fs laser: femtosecond laser; SLM: spatial light modulator; CCD: charge coupled device.

1. The data processing method

We take one of the pictures of image encryption experiments as an example to illustrate the process of original data processing, which is shown in **Figure S4**. Firstly, we code a program to scan the recording area to acquire the corresponding nonlinear upconversion luminescence intensity distribution, as shown in Figure S4(a). Then, we calculate the bit error rate corresponding to different thresholds, and select the appropriate threshold to binarize the nonlinear upconversion luminescence image, which is shown in Figure S4(b). The bit error rate (BER) can be defined as:

$e=\frac{\sum_{m} \sum_{n} \left| A_{mn}-B_{mn} \right|}{N}$ (2)

where $|A_{mn}-B_{mn}|$ denotes the error bit between the original data and the extracted binarization data of all the information units. N is the total number of information units.

For the statistical graph of the nonlinear upconversion luminescence intensity distribution of each pixel in Figure S4(a), there are two Gaussian distributions corresponding to the recorded points and the non-recorded points, as shown in Figure S4(d). Figure S4(b) shows the binarized pattern at the threshold intensity Ith = 0.2894. The bit error rate corresponding to this set of data is 0.0044 and this result demonstrate that our concept can be used for encryption with high-quality and low crosstalk.


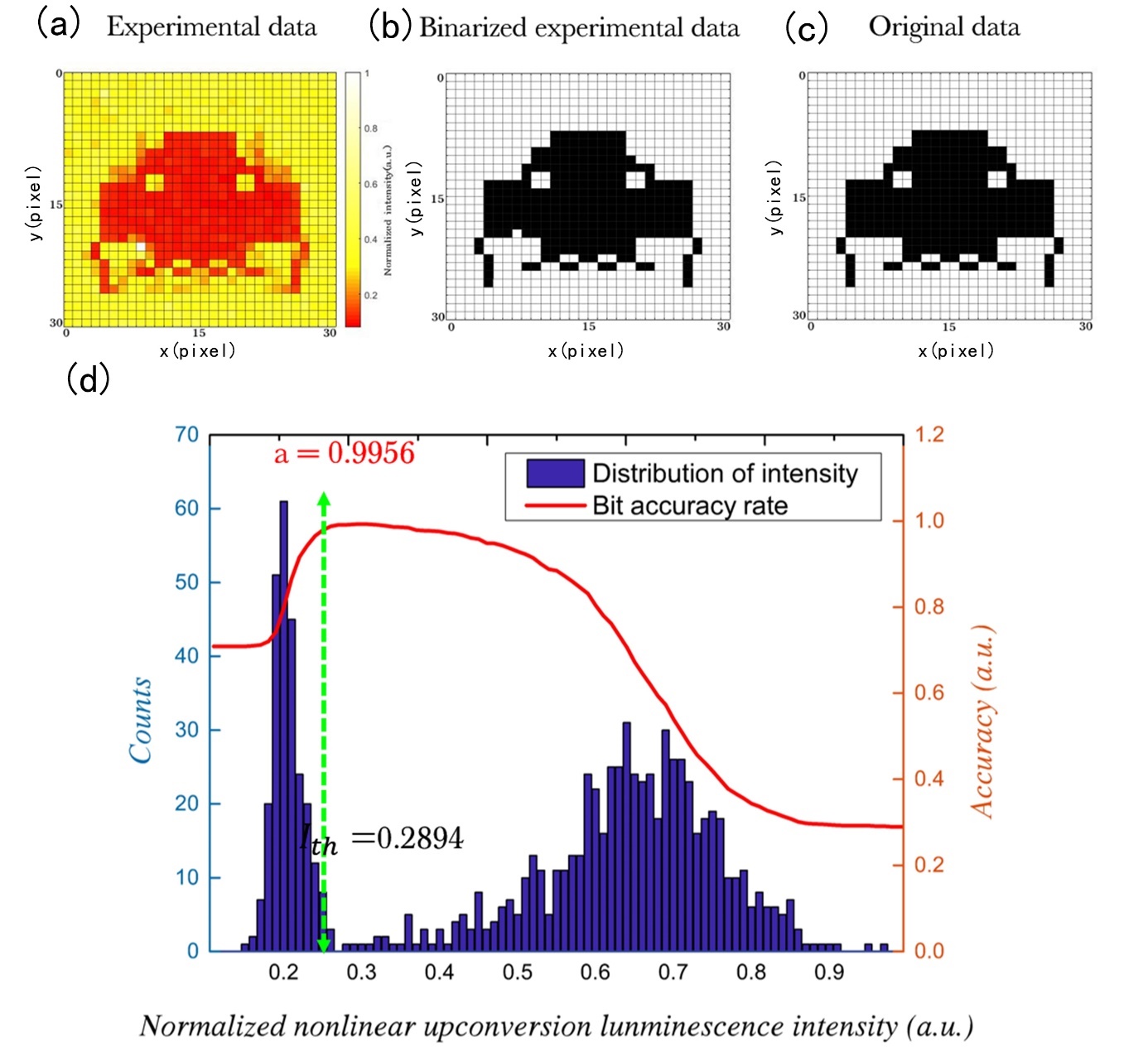


**Figure S4.** a) The pattern obtained by detecting the nonlinear upconversion luminescenceintensities of all the information units. The size of the recording region was 45 × 45 μm with 30 ×30 pixels. b) The result of binarizing the nonlinear upconversion luminescence image by selecting an appropriate threshold. c) The original pattern used for data recording. d) The distribution of the nonlinear upconversion luminescence intensities of all the information units in the recording region. The calculated fidelity (a = 0.9956) and threshold (Ith = 0.2894) for the extracted pattern is also provided.

1. The nonlinear upconversion luminescence intensity distributions


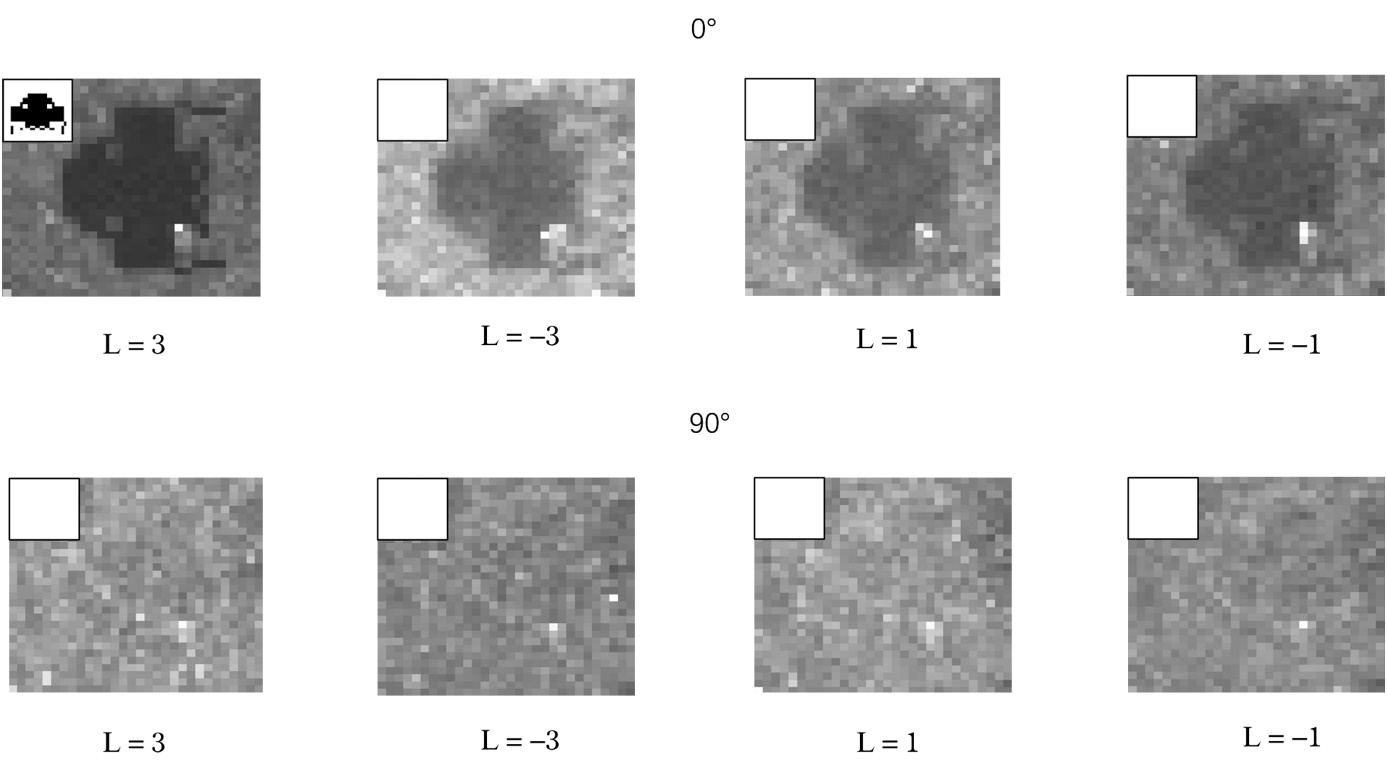


**Figure S5.** The nonlinear upconversion luminescence images corresponding to every polarization and topological charge combinations in Figure 4(c). The insets represent the reconstructed image after processing.

1. The nonlinear upconversion luminescence spectra


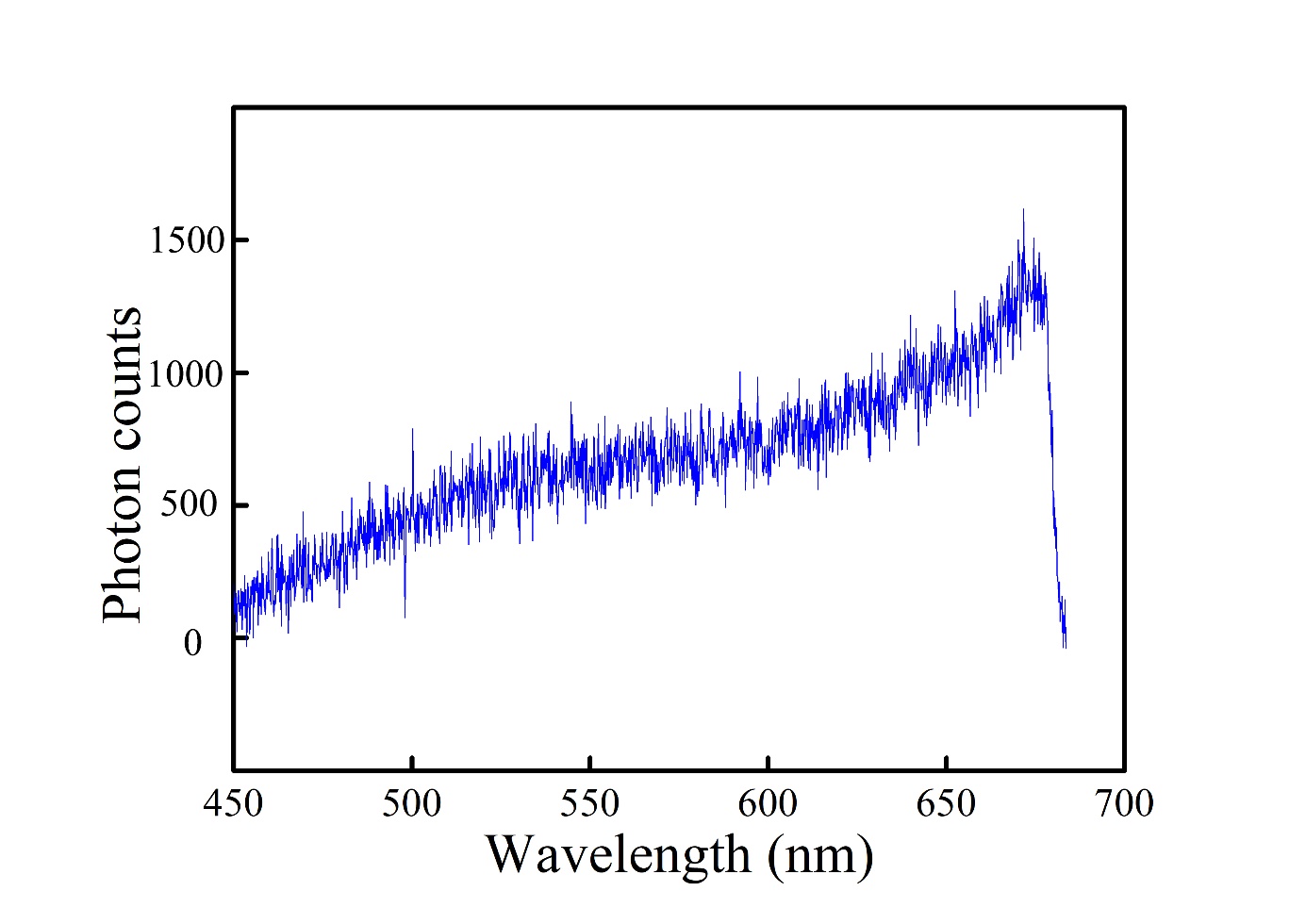


**Figure S6.** The nonlinear upconversion luminescence spectra from the gold rod samples.
